# Assessing the impact of COVID-19 passes and mandates on disease transmission, vaccination intention, and uptake: a scoping review

**DOI:** 10.1186/s12889-023-17203-4

**Published:** 2023-11-17

**Authors:** Yessika Adelwin Natalia, Margaux Delporte, Dries De Witte, Philippe Beutels, Mathias Dewatripont, Geert Molenberghs

**Affiliations:** 1 I-BioStat, Data Science Institute, Hasselt University, Hasselt, Belgium; 2 I-BioStat, Department of Public Health and Primary Care, Faculty of Medicine, KU Leuven, Leuven, Belgium; 3 Centre for Health Economics Research and Modelling Infectious Diseases, Vaccine and Infectious Disease Institute, University of Antwerp, Antwerp, Belgium; 4 I3h, ECARES and Solvay Brussels School of Economics and Management, Université Libre de Bruxelles, Brussels, Belgium

**Keywords:** COVID-19, Intention, Pass, Scoping review, Transmission, Uptake, Vaccine

## Abstract

**Purpose:**

Policymakers have struggled to maintain SARS-CoV-2 transmission at levels that are manageable to contain the COVID-19 disease burden while enabling a maximum of societal and economic activities. One of the tools that have been used to facilitate this is the so-called “COVID-19 pass”. We aimed to document current evidence on the effectiveness of COVID-19 passes, distinguishing their indirect effects by improving vaccination intention and uptake from their direct effects on COVID-19 transmission measured by the incidence of cases, hospitalizations, and deaths.

**Methods:**

We performed a scoping review on the scientific literature of the proposed topic covering the period January 2021 to September 2022, in accordance with the PRISMA-ScR guidelines for scoping reviews.

**Results:**

Out of a yield of 4,693 publications, 45 studies from multiple countries were retained for full-text review. The results suggest that implementing COVID-19 passes tends to reduce the incidence of cases, hospitalizations, and deaths due to COVID-19. The use of COVID-19 passes was also shown to improve overall vaccination uptake and intention, but not in people who hold strong anti-COVID-19 vaccine beliefs.

**Conclusion:**

The evidence from the literature we reviewed tends to indicate positive direct and indirect effects from the use of COVID-19 passes. A major limitation to establishing this firmly is the entanglement of individual effects of multiple measures being implemented simultaneously.

**Supplementary Information:**

The online version contains supplementary material available at 10.1186/s12889-023-17203-4.

## Introduction

COVID-19 passes (also referred to as immunity passports or COVID-19 certificates, among other terms) are documents granted to individuals who are vaccinated, have recently recovered from the infection, and/or have tested negative. These passes have been considered an incentive to compensate for social restrictions and as a preventive measure to allow more normal activities. Variations in the implementation exist, but in most countries, the pass would document its holder’s COVID-19 immunity status and therefore allow him or her to participate in various activities, such as cultural and sports events, conferences, or visiting tourist attractions. Israel was the first to implement this sort of incentive, known as the Green Pass, in March 2021 [1]. The Green Pass was given to Israelis who had been vaccinated with two doses of a COVID-19 vaccine, who recovered from a COVID-19 infection, or who were at that time participating in a clinical trial for vaccine development. Other countries soon followed this example. Denmark launched *Coronapas* in May 2021 for citizens who had been fully vaccinated or had received the first dose for at least two weeks, had a negative test taken within the last 72 hours, or had recently recovered from a COVID-19 infection [2]. The European Union issued the EU Digital COVID Certificate (EUDCC) in June 2021 to facilitate travel across different member states [3]. In the same month, The Netherlands invoked a similar system called the *Corona Toegangsbewijs* within the context of their pandemic response framework [4]. One month later, Italy introduced the Green Digital Pass to access work and leisure places [5]. In August 2021, French authorities implemented the *Passe Sanitaire* [6] while Germany used the “3-G (*geimpft, genesen, getestet*)” rule [7]. Belgium introduced a national COVID-19 pass under the name of Covid Safe Ticket in October 2021 [8].

The use of COVID-19 passes has been evolving rapidly in accordance with the evolution of COVID-19 cases worldwide. At the time of writing (February 2023), almost all restrictions had been lifted and the use of COVID-19 passes was no longer mandatory in many countries. However, up to this date, only a few articles discussed the impact of COVID-19 passes on COVID-19-related health indicators. To highlight the gaps in this area, we conducted a scoping review to explore the impact of COVID-19 passes on (1) the number of confirmed cases, hospitalizations, intensive care admissions, and mortality (we refer to this as the direct effects), and (2) vaccination intention and uptake, which eventually impacts the COVID-19 indicators as well (we refer to this as the indirect effects).

## Materials and methods

This scoping review was reported according to the Preferred Reporting Items for Systematic Reviews and Meta-Analyses extension for Scoping Reviews (PRISMA-ScR) [9].

### Search strategy

The search was performed on November 23, 2022, and included all relevant studies from the following databases: Pubmed, Web of Science, and Scopus. A combined set of keywords was used to search these databases. We only included results starting from January 1, 2021, since the implementation of COVID-19 passes did not start before effective vaccines were available. Considering various terms of COVID-19 passes that have been used around the world, searches with the following keywords were carried out:

- Covid Safe Ticket/Testen voor Toegang/Pass Sanitaire/Green Pass/1G/2G/3G/corona pass/corona passport/vaccin* pass/vaccin* passport/vaccin*mandate/vaccin*certificate/proof of vaccination
- Covid*/SARS*

The search queries for each database are shown in Table 1. The search was performed without any language restriction, but only studies written in English were considered. In addition, both working papers and peer-reviewed articles are included in the scoping review. We examined references of relevant articles to find additional eligible studies.

**Table 1:** Queries used to search the database

Search query	Database	Yield as of November 23, 2022
(Covid Safe Ticket OR Testen voor Toegang OR Pass Sanitaire OR Green Pass OR 1G OR 2G OR 3G OR corona pass OR corona passport OR vaccin* pass OR vaccin* passport OR vaccin* mandate OR vaccin* certificate OR proof of vaccination) AND (Covid* OR SARS*)	Pubmed	1,241
	Web of Science	1,016
ALL(“Covid Safe Ticket”) OR ALL(“Testen voor Toegang”) OR ALL(“Pass Sanitaire”) OR ALL(“Green Pass”) OR ALL(“1G”) OR ALL(“2G”) OR ALL(“3G”) OR ALL(“corona pass”) OR ALL(“corona passport”) OR ALL(vaccin* pass) OR ALL(vaccin* passport) OR ALL(vaccin* mandate) OR ALL(vaccin* certificate) OR ALL(proof of vaccination) AND ALL(Covid*) AND ALL(SARS*)	Scopus	4,013

### Study selection

We removed duplicate studies using EndNote X20. In the first round, the title and abstract of each record were reviewed independently by two authors. In the second round, the full text was retrieved and two authors independently reviewed the text. In cases of disagreement, a third author reviewed the article to reach a consensus.

### Inclusion and exclusion criteria

The inclusion criteria were (a) articles (including pre-prints) reporting the use of COVID-19 passes in a certain setting; (b) the study was not purely descriptive in nature and used well-established study designs such as cross-sectional, case-control, cohort, and clinical trials, among others. Articles without relevant content (e.g., articles discussing digital solutions for COVID-19 passes, ethical or legal considerations), articles with limited relevant content (e.g., articles only mentioning COVID-19 passes without further quantitative analysis, non-empirical studies), letters to the editor, and news reports were excluded.

### Data extraction

We extracted important key findings from the full text of each eligible study. These key findings were then compared and described using a narrative form accompanied by “summary-of-findings” tables. Even though it is not mandatory in a scoping review, we also evaluated the methodological quality of the studies included in this review using the Joanna Briggs Institute Critical Appraisal Tools for observational studies [10] and the EPIFORGE checklist for simulation studies [11]. The evidence level of each study was classified as “fair”, “moderate”, or “good” if equal to or less than 50%, $51-80\%$, and more than 80% of the items were rated as “yes”, respectively [12].

## Results

### Search and selection

**Fig. 1:**
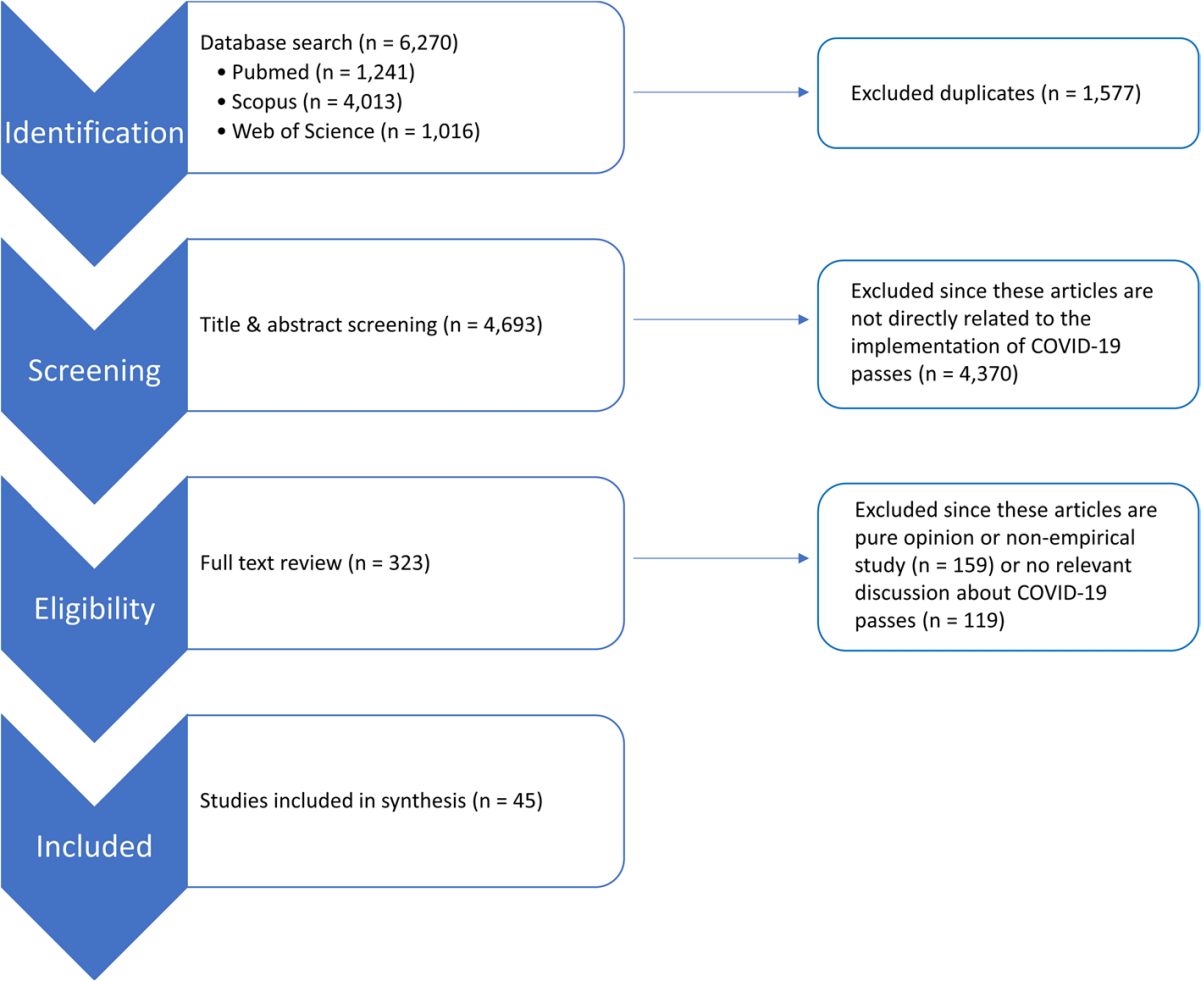
Flow diagram of literature selection

The initial search identified 6,270 records (Fig. 1), which we further reduced to 4,693 after removing duplicates as described by Bramer et al. [13]. Based on title and abstract screening, 4,370 records were excluded because these articles did not discuss the implementation of COVID-19 passes or the impact thereof. Of the remaining 323 papers, more than 80% of the articles were disqualified upon full-text consideration, mostly due to non-empirical point-of-view or inadequate discussion about COVID-19 passes. A total of 45 articles were included in this scoping review. The complete list of these studies with their key findings can be found in Tables S1 and S2.

### Characteristics of the studies

Among the 45 articles selected, 34 (75.56%) were cross-sectional studies, one (2.22%) was a cohort study, one (2.22%) was a randomized controlled study, one (2.22%) was a quasi-experimental study, and eight (17.78%) were modeling studies. Quality assessment based on the checklists showed that 24 studies (53.33%) were of moderate quality and 18 studies (40%) were of good quality as shown in Table S3. Three studies (6.67%) were considered of fair quality. The main issues that lowered study quality were unclear inclusion criteria in four studies (8.89%), no consideration of confounding factors in 11 studies (24.44%), or the results availability as a public data object in eight mathematical modeling studies (17.78%).

The selected studies came from different countries and areas. Fourteen studies (31.12%) used population data from European countries, two studies (4.44%) used Asian population data, nine studies (20%) were conducted in the African and Middle East region, 11 studies (24.44%) were conducted in North America, and five studies (11.11%) used data from multiple countries in different regions. Three studies (6.67%) used simulations to study the effect of COVID-19 passes in different situations.

Half of the studies used an online survey to collect data ($n=22$), in which participants were recruited via social media, contacts, mail, or web pages. A few studies mentioned random representative sampling (e.g., by age or sex), while others included various forms of convenience or snowball sampling. Sixteen studies (35.56%) used publicly available data. Thirty-four studies focused on the general population, while the rest explored a more specific population, the healthcare workers.

**Fig. 2:**
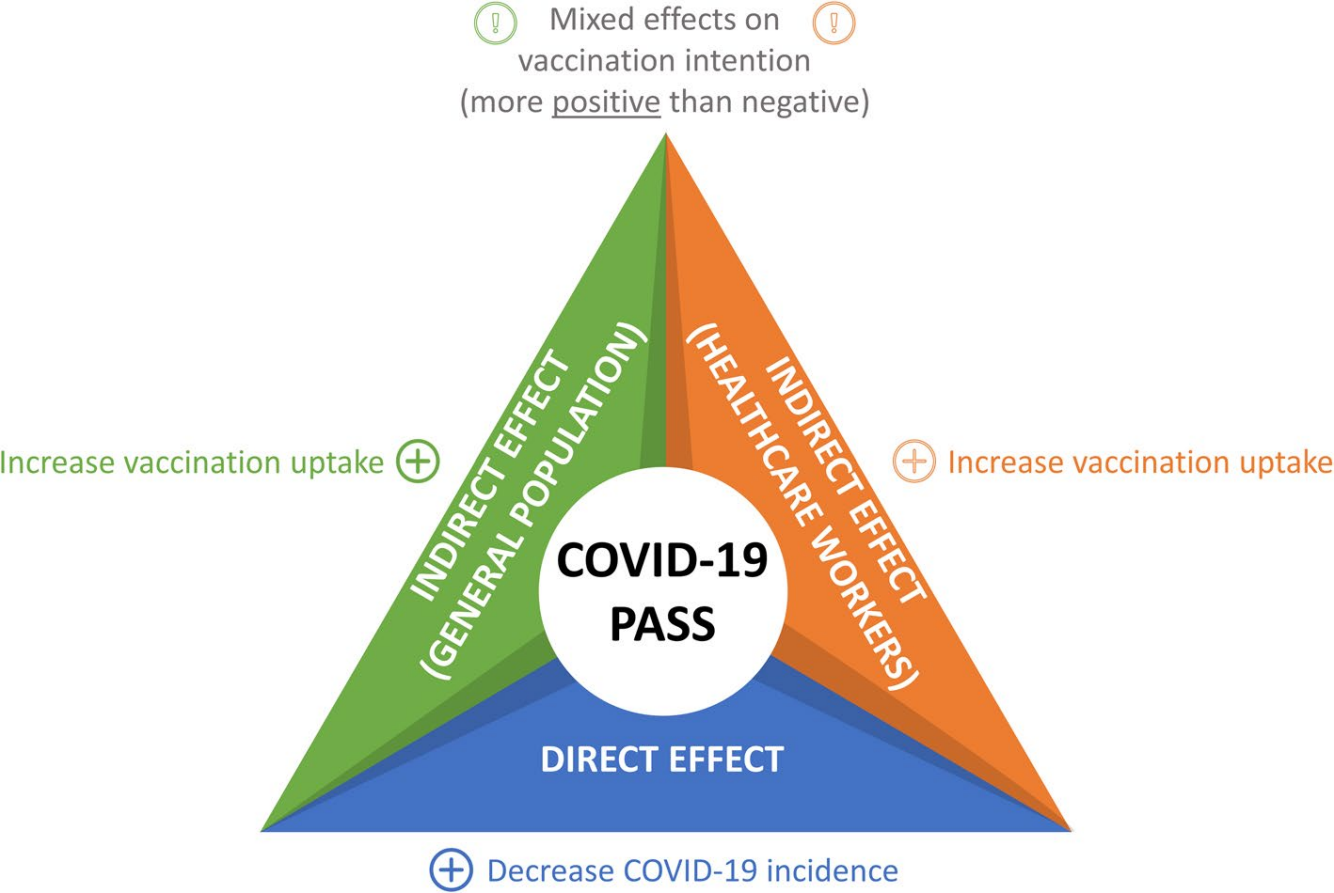
Summary of the main findings

The main findings are summarized in Fig. 2, with a comprehensive discussion provided in the following two subsections.

### Direct effect on transmission

COVID-19 passes restrict entrance to certain places or public events. In this way, the number of contacts of non-vaccinated or infected people can be reduced, which eventually directly prevents COVID-19 transmission to some extent. Only seven studies investigated the direct effect of COVID-19 passes and six of them reported similar results, in the sense that COVID-19 passes were able to reduce the number of cases and deaths. Juarez et al. [14] evaluated the impact of COVID-19 passes on infections in Hawaii by comparing the rate of new COVID-19 cases per 100,000 individuals of counties that implemented a certificate (Honolulu and Maui counties) to counties that did not implement such a certificate (Kauai and Hawaii counties). They found a significantly lower rate of new COVID-19 cases per 100,000 individuals in counties in which a COVID-19 pass was implemented. Ramos et al. [15] compared the COVID-19 incidence rate between people who attended public events that required a COVID-19 pass and people who did not attend such events in a non-randomized controlled study, conducted in Girona, Spain. The results showed that the COVID-19 incidence rate was lower in the intervention group (attendees) at 7 and 14 days of follow-up, but the difference between the intervention and control groups was not statistically significant, indicating that attendance to social events requiring a pass for access was not associated with a change in the risk of infection.

Some counties in the United States implemented vaccination mandates for colleges and universities. In the study of Ghaffarzadegan [16], the effect of this mandate was evaluated and they reported that mandating vaccination decreased cases by 1,473 cases per 100,000 student population (95% CI: 132 – 2813), indicating that a COVID-19 vaccine requirement can indeed have a positive effect to reduce the number of COVID-19 cases in higher education settings.

The study of Cuschieri et al. [17], however, reported a contrast in the context of the EURO2020 football championship, which was the first pan-European mass sports event after the onset of the pandemic. Following the EURO2020 championships, a general increase in COVID-19 cases was observed both on the local (host cities/regions) and national (participating and non-participating countries) levels. The authors suggested that the increase might be attributed to the external group gatherings that occurred on the streets, in squares, in bars, and at private events outside the stadium, which did not require COVID-19 passes.

Next to these observational studies, there were three other studies that used mathematical modeling in their analysis. Hohenegger et al. [18] developed a mathematical model to compare the effect of two types of COVID-19 passes: the first type was granted to individuals with a vaccination certificate or a recent negative test (VT-HP) and the second type was granted to vaccinated individuals (V-HP) only. They concluded that a V-HP is much more efficient in reducing the number of infected individuals. Moreover, if the reduction in the contacts by the use of V-HP reached 20–40%, it would be powerful enough to suppress a potential next wave based on the simulated model. Tchepmo Djomegni et al. [19] also argued in a simulation study that the “protected” group, which contains people who have evidence to be risk-free of being infected, i.e., people with COVID-19 pass, has a beneficial effect to reduce new secondary infections. Despite the clear positive effect of COVID-19 pass, Burgio et al. [20] added the importance of homophily, which is the premise that physical contacts are more likely to occur between individuals with similar socio-demographic and behavioral characteristics. In this context, they argued that COVID-19 passes can reduce the likelihood of contact between vaccinated and non-vaccinated individuals and this effect on the mixing rate of vaccinated and non-vaccinated individuals should not be overlooked. When this effect is not taken into account, the effect of the COVID-19 pass is potentially overestimated.

### Indirect effect on vaccination

Forty-one studies discussed the indirect effect of COVID-19 passes on vaccination. We distinguish the effect on vaccination intention and vaccination uptake. Vaccination intention is a psychological construct and refers to the willingness of an individual to get vaccinated. It is important to note that vaccination intention is a strong predictor of actual vaccine uptake [21], but eventually, vaccination uptake, which is the proportion of a population that has been vaccinated at a certain time point, reflects more the act of getting vaccinated at the group level. We further discussed the indirect effect in two subgroups: the general population and the healthcare workers (including students in healthcare-related fields)

#### Vaccination intention in the general population

The study of Albarracin et al. [22] found that intentions to get vaccinated were significantly higher when vaccination was required than in the freedom of choice condition. Therefore, they conclude that requiring the vaccine via a vaccination mandate could increase vaccination intentions. However, it is important to note that in this study, the effect of a vaccination mandate was investigated instead of a general COVID-19 pass. Moreover, the use of this mandate was also stricter than passes such as the Green Pass; under the required vaccination condition, COVID-19 vaccination was also required to work or go to school.

Other studies in Saudi Arabia [23], the United Kingdom [24], as well as many other countries [25–30] reported similar positive effects of incentives in the form of a COVID-19 pass on willingness to get vaccinated. On top of that, potential mandatory vaccination to get a COVID-19 pass could drive the likelihood of accepting the COVID-19 vaccine, particularly when it is related to travel requirements [31, 32]. However, an important remark is that some studies used multiple incentives including COVID-19 pass as their exposure since multiple facilitation measures are needed to encourage vaccination intention and uptake [29, 32].

Nevertheless, some other studies reported negative effects of COVID-19 passes. Porat et al. [33] explored how people’s willingness and motivation to get vaccinated depends on their psychological needs (autonomy, competence, and relatedness), and how a COVID-19 pass might affect these needs. They found that need frustration, i.e., when basic psychological needs of autonomy, competence, and relatedness are being undermined due to social influences, is associated with lower willingness to get vaccinated, and that the implementation of COVID-19 pass might lead to greater need frustration. Shmueli [34] found no significant association between the Green Pass and the sense of urgency to receive the vaccine, indicating that implementing the Green Pass would not increase the intention to receive the vaccine immediately. In a discrete choice experiment conducted by Mouter et al. [35], the respondents showed a negative preference for policies that promote vaccination, particularly if these policies punish those who reject vaccination. Even though Okamoto et al. concluded that vaccine passports could indeed increase vaccine acceptance in Japan, we should also note that the study was based on a hypothetical conjoint experiment [36]. The study of Sargent et al. reported 32.1% of the working unvaccinated participants would get vaccinated if their work required it, 42.2% would not get vaccinated and 25.7% said they were unsure. This suggests that there were mixed reactions toward work mandates among the participants who are resistant to vaccination [37].

#### Vaccination uptake in the general population

In contrast to the vaccination intention, we found more consistent results, i.e., authors reported positive influences on the vaccination uptake.

Israel was the first country that implemented a COVID-19 pass. After the implementation of the Green Pass, Saban et al. [38] studied the patterns of COVID-19 vaccination in Israel. Their study suggested that the Green Pass increased vaccination uptake, although a causal relationship could not be established. They also suggest that incentives like the Green Pass are more likely to influence younger people. Similar results were reported in the study of Juarez et al., who found an increase of 1.41% in vaccination uptake in counties that implemented a COVID-19 pass [14]. Oliu-Barton et al. [39] reported an increase in vaccination uptake of 13.0, 10.7, and 6.2 percentage points in France, Italy, and Germany, respectively, which led to averted new cases and deaths. By introducing COVID-19 passes, an additional 32,065 hospital admissions were estimated to have been prevented in France, 5,229 in Germany, and 8,735 in Italy. There would have been 3,979 additional deaths in France, 1,133 deaths in Germany, and 1,331 deaths in Italy if the COVID-19 pass would not have been implemented. Nevertheless, the authors emphasize that a causal relationship cannot be inferred directly from their analysis and that the magnitude of the impact is very different between countries due to varying factors (e.g., the way in which COVID-19 passes were implemented). Similar favorable results were reported in different settings [26, 40–47].

In the settings where the vaccination mandate was implemented, Bennett et al. found a significant increase in vaccination uptake by 8.7 percentage points at some workplaces [44]. Howard-Williams et al. compared 13 state-level jurisdictions with a vaccine mandate (without a test-out option) to 14 state-level jurisdictions that allowed a test-out option and/or did not issue any type of mandate. They found a higher percentage of the population receiving the first dose of the vaccine in the jurisdictions with a vaccine mandate [48]. Cohn et al. investigated the joint impact of a proof-of-vaccination requirement, incentive payments, and employer-based mandates on the rates of adult vaccination in New York City. They reported a larger increase (+16.9 percentage points) compared to jurisdictions that did not implement this combination. However, it was difficult to distinguish the true effect of the vaccination mandate since they studied the combination of these three incentives [45].

Additional factors should also be considered when interpreting these results. In the study of Mills and Rüttenauer [40], the effectiveness of a COVID-19 pass depends on the pre-existing levels of vaccination, i.e., in countries with a vaccination uptake below the average of the synthetic control group included in the study, the increase in vaccine uptake was higher compared to countries with an average to high vaccination uptake. Reno et al. stressed that the changes in vaccination uptake (and eventually COVID-19 indicators) might not be attributed to a COVID-19 pass, but rather to the other non-pharmaceutical interventions (NPIs) (e.g., social distancing, face mask) that were still in vigor [42]. Karaivanov et al. [43] argued that the variation in COVID-19 pass inside and outside Canada indicates other factors (e.g., announcement timing, percentage of unvaccinated people) might have an influence. The study of Saban et al. and Kluver et al. reported that a COVID-19 pass would not have any effect on individuals who are against vaccinations on principle, e.g. those with personal, cultural, or religious beliefs that discourage them from vaccinating [26, 38].

#### Vaccination intention in healthcare workers

Similar to the general population, there are many factors affecting vaccination intention. However, it should be noted that vaccination among healthcare workers was of a different nature compared to that in the general population, given that some countries implemented vaccine mandates for this subpopulation.

Most studies found factors that positively influence vaccination intention. Being female, having a higher income level, being married, and having a higher level of education significantly impact COVID-19 vaccine acceptance among Saudi Arabian healthcare workers [49]. On top of this, Iwu et al. found that healthcare workers who have a tendency to encourage patients to take vaccines, have trust in the government, or feel they would receive the vaccine if their friends have all been vaccinated would be less hesitant to accept COVID-19 vaccination [50]. Other factors were reported by Hubble et al. [51] where vaccine safety and effectiveness, the importance of vaccination to protect patients, perceived personal risk of infection, previous acceptance of influenza vaccine, and sufficient knowledge to make an informed decision about vaccination positively influence the vaccination intention among the emergency medical service professionals in the United States.

Kaufman et al. found higher intention to be vaccinated among primary healthcare workers, men, medical doctors, older age groups, full-time employees, or those living in major cities. More than half (57%, 1754/3058) said they would be more likely to get vaccinated if required by their employer [52]. Ledda et al. reported different acceptance rates of mandatory vaccination by different vaccine-preventable diseases, but it increased considerably during the COVID-19 pandemic. Higher acceptance of mandatory vaccinations was expressed by healthcare workers caring for immunocompromised patients [53]. Another study from Maltezou et al. found that being male, being a physician, being completely vaccinated against hepatitis B, having been vaccinated against H1N1 during the pandemic in 2009-2010, holding a belief that COVID-19 vaccination should be mandatory for health care professionals, and having higher confidence in vaccines in general during the ongoing COVID-19 pandemic were positively associated with intention to get vaccinated. On top of this, 776 (49.8%) of the participating healthcare workers favored mandatory vaccination policies [54].

Another perspective came from students in healthcare-related fields. Kelekar et al. conducted a study among medical and dental students in the United States. They found that students who thought the COVID-19 vaccine was important to them as healthcare workers, trusted COVID-19 information received from public health experts, and thought the COVID-19 vaccination should be mandatory for the general public were more likely to report willingness to get the COVID-19 vaccine after controlling for demographic variables, experience with COVID-19, and personal vaccination behaviors [55]. On top of this, potential mandatory vaccination to get a COVID-19 pass could drive the likelihood of accepting the COVID-19 vaccine among medical students, particularly when it is related to travel requirements [56].

Despite these positive influences on vaccination intention, some studies found negative results. For example, Peruch et al. reported that 17.7% of the participating healthcare workers did not agree with the vaccine mandate even though the vaccination rates were high in Italy. Around 5.4% stated that they agreed to be vaccinated exclusively because of the sanctions given by the legislation [57]. Hubble et al. also reported that only 18.7% supported mandatory vaccination for emergency medical service professionals [51]. In Nigeria, more than half of the healthcare workers would not encourage vaccination mandates [50]. Arif et al. found high vaccine hesitancy among Saudi Arabian healthcare workers and that COVID-19 vaccination mandates decreased the odds ratio of vaccine acceptance by 0.27 [49].

#### Vaccination uptake in healthcare workers

McGarry et al. [58] conducted a study to assess the association between state vaccine mandates and the vaccination rates among nursing home employees in the United States. They found an increase in the mean staff vaccine coverage by 5.4 percentage points (95% CI, 1.1–9.8) in mandate states without a test-out option and 2.2 percentage points (95% CI, 0.8–3.5) in mandate states with a test-out option following mandate announcement. Similar results were reported by Syme et al. [59]; the staff COVID-19 vaccination rates in Mississippi increased from 43.0% before the vaccinate-or-test-out mandate to 51.3%. Thus, similar to the general population, there is a positive effect of the COVID-19 mandate on vaccination uptake.

## Discussion

In this scoping review, we aimed to investigate the effect of the use of COVID-19 passes on several COVID-19 indicators (infections, hospitalizations, and mortality), which we refer to as the direct effect, and vaccination, which we refer to as the indirect effect. A total of 45 studies were included in this review. We discussed the results further in the following subsections.

### Direct effect on transmission

A limited number of studies examined the direct effect of COVID-19 passes on COVID-19 indicators. Three observational studies concluded that COVID-19 passes decreased the number of new cases, hospitalizations, and/or deaths. In contrast with these results, another study was less optimistic about COVID-19 passes and noted that the use of these passes in a large closed event could not prevent external group gatherings outside the event. Three other studies incorporated mathematical models in the context of certification. One study directly modeled the effect of implementing COVID-19 passes, if we assume that individuals who are granted a pass are non-infectious [19]. They concluded that investing in individuals with no risk of infection is a better strategy compared to other strategies such as having more recovered individuals (i.e., herd immunity). Another study compared two variants of COVID-19 passes and favored the variant where a COVID-19 pass is given to vaccinated individuals only, in contrast with a pass that is handed to individuals who are vaccinated or recently tested negative [18]. The third study did not model the direct effect of certification explicitly but stressed that the direct effect can be overestimated due to the effect of COVID-19 passes on the mixing rate between vaccinated and unvaccinated individuals. Despite this limited evidence, we can conclude that COVID-19 passes reduced COVID-19 transmission to some extent. It should be noted that there is a gap in the literature concerning mathematically modeling the direct effect of COVID-19 passes on transmissions, most likely due to limited data to determine model parameters related to the COVID-19 pass effect on behavior and transmission. The studies we included often relied on unrealistic assumptions, for instance, that all individuals with a COVID-19 pass are non-infectious all of the time.

### Indirect effect on vaccination in the general population

Even though there was a lot of debate about the implementation of COVID-19 passes [60–63], most studies in this review concluded that COVID-19 passes have a positive effect on vaccination uptake and vaccination intention, indicating that more people are likely to get vaccinated when COVID-19 passes are implemented. However, this positive effect requires appropriate nuancing. Some studies found that vaccine uptake was only increased among individuals who already intended to get vaccinated, regardless of whether or not COVID-19 passes are granted. Among individuals who do not intend to get vaccinated, the implementation of COVID-19 passes had no effect. Similar results were reported by Batteux et al. [64] and Drury et al. [65]. Mills and Rüttenauer also argued that the effect depends on the pre-intervention vaccination uptake [40]. Another key finding of our review is that a number of studies found a higher vaccine hesitancy among older people and a larger effect of COVID-19 passes on vaccine uptake in younger age groups compared to older age groups. Vaccine hesitancy in the older age group was commonly reported in different settings [21, 66, 67]. Despite this hesitancy, COVID-19 vaccination uptake was still high among the elderly population, especially nursing home residents or elderly people who are familiar with new technologies (e.g., online vaccination appointments, QR code for COVID-19 pass) [68].

### Indirect effect on vaccination in healthcare workers

We expected higher vaccination rates and acceptance among healthcare workers since they are well-educated in health. While the studies in our synthesis reported high vaccination rates in this subpopulation, we also found hesitancy and even to some extent, rejection of the vaccination mandate. Similar to our findings, low support for COVID-19 vaccine mandates was found in Nigeria [69], France [70], and Cyprus [71]. The most common reasons for this hesitancy were related to the safety of this “new” vaccine and also some distrust in the government or the reported results from large-scale randomized trials [72]. The decision to be vaccinated is certainly influenced by many factors. However, an important factor that needs to be considered is the information conveyed to the public. We should note that healthcare workers consist not only of medical professionals (doctors, nurses, paramedics), but also technicians, pharmacists, healthcare assistants, and other healthcare professionals who rely more on other sources of information such as the internet, social media, news, family, or friends [73]. Therefore, it is important to provide the right information to the general public as well as a more specific population such as healthcare workers.

### Strengths and limitations

The strength of our study lies in the utilization of various electronic databases with search strings tailored to various terms of COVID-19 passes. The articles were reviewed and controlled by three independent people. On top of this, we presented the methodological quality of each study using well-established critical appraisal tools.

There are several limitations that should be considered. First, the implementation of COVID-19 passes was always accompanied by other NPIs such as the use of face masks or social distancing. Some countries also implemented several other incentives on top of the COVID-19 pass to boost vaccine uptake. It is therefore difficult to separate the effect of the COVID-19 pass and infer a causal relationship. However, we can conclude that these measures (and therefore, the COVID-19 pass itself) contributed to preventing COVID-19 transmission. Moreover, most studies tried to overcome this limitation by using similar countries or regions with a similar trend before a COVID-19 pass was implemented as counterfactuals or by using counterfactual simulations. Second, most of the studies included in this scoping review investigated the implementation of COVID-19 passes before the Omicron variant emerged in November 2021 [74]. This new variant changed the transmission dynamics in the population and reduced the impact of preventive measures such as COVID-19 passes on the transmission. Nevertheless, these results remain relevant to be considered for future reference.

Third, although we searched for studies without limitations in study designs, this scoping review included mostly cross-sectional or modeling studies. Thus, it only allows us to draw inferences regarding association and to a very limited extent, causality. Fourth, we searched only the primary databases for published research in medical and to some extent, non-medical sciences. Therefore, there is a possibility that publications and reports that are not indexed in these databases were excluded. These choices were guided by the opportunity to use complex queries due to different terms and variations of COVID-19 passes used around the world. On top of this, we were interested in the most relevant and arguably the highest-quality research that has been peer-reviewed.

Lastly, one could argue that a COVID-19 pass can also have an effect on behavior. For instance, it might be possible that individuals who are not in possession of a COVID-19 pass are less likely to go to events or public places that require a pass but perhaps go to illegal events or places that do not check for passports. On the other hand, individuals who obtain a COVID-19 pass can experience a false sense of security, leading to more risky behavior. Studying these behavioral effects of a COVID-19 pass was not part of our research, nor was a consideration of the ethical and social implications.

### Future research and suggestions

Clearly, it will be of interest for future pandemic planning to investigate the potential impact of COVID-19 (and eventually other infectious diseases) passes on transmission, disease, and vaccine uptake, along with its potential negative consequences on public trust in times of crisis. In order to improve our understanding of the mechanisms involved, the influence of such passes on the intricacies of human behavior should be part of such investigations.

Our study underscores the favorable implications of COVID-19 passes. Nevertheless, the implementation of COVID-19 passes is a complex policy decision that requires balancing various factors, including public health, individual rights, and economic aspects. Since they are by definition a measure subject to temporary use, “infectious disease passes” could be considered in the first place to bridge epidemic periods during which unbridled social contacts in some parts of the economy can damage other (much larger) parts of the economy, as well as public health and health care system functioning. In this regard, policymakers are encouraged to meticulously assess the contextual nuances inherent to their respective jurisdictions while maintaining an overarching commitment to the preservation of public health and safety. It is also important to convey a clear rationale for the implementation of COVID-19 passes based on robust scientific evidence.

While our findings reveal that the introduction of COVID-19 passes increased vaccine uptake, it concurrently bears the potential to exacerbate vaccine hesitancy. In light of this, substantial investments in public education campaigns emerge as imperative measures to counter misinformation and foster enhanced vaccine literacy within the population.

Important lessons learned, potentially of use to policymakers, are as follows. First, there is evidence that COVID-19 passes have the potential to reduce the number of cases, hospitalizations, and deaths, in a direct way, via avoided contacts, and in an indirect way, by increasing vaccination intention and/or uptake. This potential is realized in some countries and regions studied. Although the COVID-19 pass may be effective for a substantial portion of the population, it is unlikely to have an impact among individuals who already hold strong anti-COVID-19 vaccine beliefs. Second, a massive event such as EURO2020 underscores that, even though passes may apply at the core events (such as sports games), they may not at associated gatherings (in restaurants, bars, public transport, etc.). Via such massive events, cases will inevitably rise, and passes will dampen but not avoid that phenomenon. Third, restriction to passes that exclude testing as an option may be more effective, as they further reduce mixing between vaccinated/recovered individuals and others. Fourth, positive effects on the intention to get vaccinated have been found, but not uniformly so. Some authors found that it can even have a negative effect, especially if there are unfavorable consequences associated with non-vaccination. Fifth, the effect on actual vaccination uptake is generally favorable, even when the effect is not so clear on the intention. Also here, the effect is virtually absent in individuals and communities with strong (a priori) anti-vaccination ideas. Sixth, both vaccination intention and actual vaccination are high to begin with in health care workers, although there are differences between subgroups. Intentions are higher in primary health care workers, males, medical doctors, city dwellers, and older people, as well as in those caring for immunosuppressed patients. That said, even people who are a priori favorable towards vaccination may take issue with a mandate. In this population, the favorable effect of a COVID-19 pass without a test-option was seen as well.

## Conclusion

To conclude, the results showed that COVID-19 passes have positive direct and indirect effects. However, the implementation should be monitored carefully since COVID-19 passes could affect many other aspects of our daily life. Follow-up retrospective analyses and reviews related to this topic should be conducted.

### Supplementary Information

**Additional file 1.**

## Data Availability

All data generated or analyzed during this study are included in this published article and its supplementary information files.
