# Supplementary material for: Assessing the impact of COVID-19 passes and mandates on disease transmission, vaccination intention, and uptake: a scoping review
Source: BMC Public Health. 2023 Nov 17;23:2279. doi: 10.1186/s12889-023-17203-4 (PMC10656887; doi:10.1186/s12889-023-17203-4)
Supplement: Supplementary file 1 — Additional file 1. [file 12889_2023_17203_MOESM1_ESM.zip › S1 Table.docx]

**S1 Table. List of empirical studies (n = 45) included in the present scoping review.**

| **Study** | **Objective** | **Context** | **Sampling** | **Methods of analysis** |
| --- | --- | --- | --- | --- |
| Albarracin et al. (2021) | To assess vaccination intentions among general and individuals with a predisposition to experience psychological reactance. | U.S. | Online survey using 4 different set of questionnaires.   - Study 1: n = 299, adult participants were recruited via Prolific. - Study 2: n = 359, adult participants were recruited via Mechanical Turk. - Study 3: n = 357, adult participants were recruited via Mechanical Turk. - Study 4: n = 606, adult participants were recruited via Qualtrics. | Descriptive analysis. |
| Alshahrani et al. (2021) | To assess the factors that could influence the acceptance and hesitancy toward the COVID-19 vaccine. | Saudi Arabia. | Online survey (n = 758, adult participants were recruited with snowball sampling). | Descriptive analysis, binary logistic regression. |
| Arif et al. (2022) | To determine the factors that can predict healthcare workers’ acceptance of the COVID-19 vaccine. | Saudi Arabia | Online survey (n = 529, adult participants were recruited from King Saud University Medical City, Riyadh). | Descriptive analysis, binary logistic regression. |
| Bennett et al. (2022) | To investigate the factors underlying COVID-19 vaccine and booster hesitancy and the efficacy of various incentives or disincentives to expand uptake. | U.S. | Online survey (n = 3,497, adult participants were recruited via Qualtrics). | T-test, multinomial logistic regression. |
| Burgio et al. (2022) | To analyze the impact of homophily (contact among individuals with similar socio-demographic and behavioural characteristics) related vaccine adoption on COVID-19 dynamics. | Simulation. | No validation. | Compartmental model: standard susceptible-infected-recovered (SIR). |
| Cohn et al. (2022) | To assess the joint effect of a proof-of-vaccination requirement, incentive payments, and employer-based mandates on rates of adult vaccination. | U.S. | Publicly available data. | Synthetic control method. |
| Cuschieri et al. (2022) | To explore the possibility of an increased general COVID-19 spread across host cities/regions, participating and neighboring countries following the first organized mass sport event. | Participants of EURO2020. | Publicly available data. | Descriptive analysis. |
| de Figueiredo et al. (2021) | To establish whether vaccine passports are likely to encourage or discourage uptake of COVID-19 vaccines among people who have not yet had two doses of a COVID-19 vaccine. | UK. | Online survey (n = 16,527, adult participants were recruited via ORB (Gallup) International panel). | Multilevel regression and post-stratification. |
| De Giorgio et al. (2022) | - To determine psychological relapses of COVID-19 booster vaccine. - To identify the determining factors affecting willingness to receive COVID-19 vaccine. - To study the relationship among emotional characteristics (anxiety, stress, depression, optimism), social media information, and the mandatory political choices (i.e., green-pass). | Croatia. | Online survey (n = 1,003, adult participants were recruited with snowball sampling). | Binary logistic regressions, cluster analysis with the k-means algorithm. |
| Dube et al. (2022) | To explore the impact of incentives and disincentive strategies on the intention to be vaccinated against COVID-19. | Quebec, Canada | Online survey (n = 8,911, adult participants were recruited via Leger). | Descriptive analysis. |
| Ghaffarzadegan (2022) | To empirically investigate the effect of the mandate policy on Fall 2021 COVID-19 cases in institutions of higher education. | U.S. | Publicly available data of selected colleges in 10 distinct states. | Difference-in-differences analysis. |
| Hohenegger et al. (2022) | To develop a simple and economical mathematical model that allows to analyze the impact of different versions of health passes on the epidemiological dynamics of an entire wave of a pandemic. | Simulation. | Validation: Germany, Austria, France, Italy and Denmark. | Compartmental model: basic and modified susceptible-infectious not vaccinated-infectious vaccinated-recovered-vaccinated (SIIRV), epidemiological renormalization group. |
| Howard-Williams et al. (2022) | To examine the association between announcing state-issued COVID-19 vaccine mandates that did not provide a test-out option for workers and the vaccine administration rates in terms of state-level first-dose vaccine administration and series completion coverage. | U.S. | Publicly available data. | Linear regression. |
| Hubble et al. (2022) | To document vaccination rates of EMS professionals and identify predictors of vaccination uptake. | U.S. | Online survey (n = 860, adult participants were recruited via Qualtrics). | Multivariable logistic regression. |
| Iwu et al. (2022) | To assess the prevalence and predictors of COVID-19 vaccine hesitancy among health care workers in tertiary health care institutions. | Nigeria. | Online survey (n = 347, adult participants were recruited from Imo State University Teaching Hospital and Federal Medical Centre in Orlu and Owerri zones). | Descriptive analysis, chi-square. |
| Juarez et al. (2022) | To evaluate the degree to which vaccine or test passport mandates impacted businesses and COVID-19 mitigation efforts. | Hawaii. | Unpublished data from The Department of Health + other publicly available data. | Descriptive analysis, difference-in-difference regressions. |
| Karaivanov et al. (2022) | To evaluate the impact of government mandated proof of vaccination requirements for access to public venues and non-essential businesses on COVID-19 vaccine uptake. | Multicountry. | Publicly available data. | Time-series analysis. |
| Kaufman et al. (2022) | To identify COVID-19 vaccine intentions, factors associated with uptake and information needs for healthcare workers. | Australia. | Online survey (n = 3,224, adult participants were recruited via RedCap). | Logistic regression. |
| Kelekar et al. (2021) | To assess COVID-19 vaccine hesitancy among medical and dental students. | U.S. | Online survey (n = 415, adult participants were recruited via Qualtrics). | Logistic regression. |
| Klüver et al. (2021) | To assess the effectiveness of three strategies to increase vaccine uptake, namely, providing freedoms, financial remuneration, and vaccination at local doctors. | Germany. | Online survey (n = 20,500, adult participants were recruited via Respondi). | OLS regression with individual level fixed effects and heteroskedasticity-robust standard errors + causal forests approach. |
| Kuznetsova et al. (202) | To assess the effect of mandatory and incentive-based vaccination measures on vaccine uptake for COVID-19. | Multicountry. | Publicly available data. | Interrupted Time Series with autoregressive integrated moving average (ARIMA) approach, counterfactual analysis. |
| Ledda et al. (2021) | To assess the knowledge and attitudes of healthcare professionals about vaccine preventable diseases before and during the COVID-19 pandemic, estimate their intention to get vaccinated against COVID-19, and search for determinants that may influence their choice. | Italy | In-person survey in two periods: before pandemic (n = 1,323) and after pandemic (n = 787) | Descriptive analysis, chi-square. |
| Maltezou et al (2021) | To estimate the level of intention to get vaccinated against COVID-19 and to explore factors that drive vaccine decision-making in healthcare workers. | Greece. | In-person survey (n = 1,571, adult participants were recruited from eight tertiary-care hospitals). | Logistic regression. |
| McGarry et al. (2022) | To assess whether state vaccine mandates for nursing home employees are associated with staff vaccination rates and reported staff shortages. | U.S. | Publicly available data. | Difference-in-differences (DID) model. |
| Mills and Rüttenauer (2022) | To investigate the effect of certification on vaccine uptake. | Treatment countries (Denmark, Israel, Italy, France, Germany, and Switzerland) versus 19 control countries. | Publicly available data. | A synthetic control method, which constructs a synthetic control country for each country that introduced mandatory COVID-19 certification. |
| Moccia et al. (2022) | To investigate vaccination hesitancy and the underlying reasons, as well as any changes to the membership following the obligation of the Green Pass. | Italy. | Online survey (n = 83, adult participants were recruited with snowball sampling). | Descriptive analysis. |
| Mouter et al. (2022) | To investigate public preferences for nine policies to promote vaccination and to examine whether preferences differ among subgroups in the population. | The Netherlands. | Discrete choice experiment (n = 747, adult participants were recruited via Kantar Profiles). | Panel mixed logit models with hierarchical Bayes technique. |
| Mustapha et al. (2021) | To investigate the factors associated with COVID-19 vaccine acceptance among health sciences students. | Nigeria. | Online survey (n = 455, adult participants were recruited via social media platforms). | Logistic regression. |
| Okamoto et al. (2022) | To assess the determinants of vaccine hesitancy, reasons for hesitation and potential effectiveness of vaccine passports used to relax public health restrictions on mitigating vaccine hesitancy. | Japan. | Online survey (n = 5000, adult participants were recruited via Cross Marketing Inc). | Multilevel mixed- effects logistic regression. |
| Oliu-Barton et al. (2022) | To measure the impact of COVID-19 certificates on vaccine uptake, health outcomes, and the economy. | France, Germany, and Italy. | Publicly available data. | Counterfactual analysis (i.e., by modelling vaccine uptake without this intervention) using innovation diffusion theory. |
| Peruch et al. (2022) | To identify the attitudes of healthcare workers related to COVID-19 vaccines, study the phenomenon of vaccine hesitancy (without focusing on a specific type or brand of COVID-19 vaccine), and evaluate opinions on the mandatory vaccination of healthcare workers. | Italy. | Online survey (n = 130, adult participants were recruited from a center for maternal and child health). | Descriptive analysis, chi-square. |
| Porat et al. (2021) | To investigate whether people’s willingness and motivation to get vaccinated depends on their psychological needs, and how vaccine passports might affect these needs. | UK and Israel. | Online survey (n = 1,358, adult participants were recruited via Prolific in the UK and PanelView in Israel). | Linear regression for continuous outcome and logistic regression for binary outcome. |
| Radic et al. (2022) | To distinguish the underlying mechanisms that could predict individuals’ intentions to take the COVID-19 vaccine as a precondition for international travel. | Multicountry. | Online survey (n = 1,188, adult participants were recruited with purposive sampling). | Covariance-based structural equation modeling (CB-SEM) with a maximum likelihood (ML) estimation method. |
| Raja et al. (2022) | To determine the acceptance and hesitancy of the COVID-19 vaccine and associated factors among medical students. | Sudan. | Online survey (n = 281, clinical phase medical students were recruited with random sampling). | Descriptive analysis, logistic regression. |
| Ramos et al. (2022) | To analyze whether people who attended public events with COVID-19 pass presented a higher rate of detected SARS-CoV-2 infections compared to a group of people who did not attend such events. | Girona (Spain). | Non-randomised controlled study (n = 5,401). | Descriptive analysis, survival analysis. |
| Reno et al. (2022) | To describe the impact of vaccine rollout and health policies on the evolution of the COVID-19 pandemic. | Italy. | Publicly available data. | Time-trend analysis of new COVID-19 confirmed cases, hospitalized patients and deaths. |
| Rosen et al. (2021) | - To describe and analyze the vaccination uptake through the end of March 2021. - To identify behavioural and other barriers that likely affected desire or ability to be vaccinated. - To describe the efforts undertaken to overcome those barriers. | Israel. | Unpublished data from The Ministry of Health + publicly available data. | Time-trend analysis. |
| Saban et al. (2021) | To examine patterns of COVID-19 vaccination and how these relate to different proposals made about benefits for those vaccinated, and to present the legal and ethical dilemmas surrounding these issues. | Israel. | Unpublished data from The Ministry of Health + publicly available data. | Time-trend analysis. |
| Sargent et al. (2022) | To investigate public reactions to these efforts to increase vaccination, including self-reported responses to widespread reduced masking behaviour, monetary incentive programs to get vaccinated, and work vaccination requirements. | U.S. | Online survey (n = 14,152, adult participants were recruited via Real-Time Interactive WorldWide Intelligence). | Chi-square tests, logistic regression, and correlational analyses. |
| Shmueli (2022) | To assess the role of incentives beyond sociodemographic, health-related and behavioural factors, in predicting the public’s intention to get COVID-19 vaccine. | Israel. | Online survey (n = 461, adult participants were recruited with convenience sampling). | Four-step hierarchical binary logistic regression, multinomial logistic regression. |
| Syme et al. (2022) | To examine whether COVID-19 vaccine mandates that allow a test-out exemption for nursing home staff are associated with increased staff vaccination rates in nursing homes. | U.S. | Publicly available data. | Time-trend analysis |
| Tchepmo Djomegni et al. (2021) | To assess the impact of a gradual, post-lockdown context concerning the spread of the disease. | Simulation. | Validation: South Africa. | Compartmental model: susceptible (S), susceptible protected (SP), asymptomatic infected not in quarantine (IA), symptomatic infected not in quarantine (IS), quarantine (Q) and recovered (R). |
| Walkowiak et al. (2021) | To compare the effectiveness of vaccination programs in two countries measured by the respective percentages of the vaccinated population. | Lithuania, Poland. | Unpublished data from The Ministry of Health + publicly available data. | Time-trend analysis. |
| Wong et al. (2021) | To examine the uptake and acceptance rate of different COVID-19 vaccines and investigate the facilitators and barriers of choices and willingness to receive COVID-19 vaccines. | Hong Kong. | Population-based telephone survey (n = 1,195, adult participants were recruited with random sampling). | Descriptive analysis. |
| Zimand-Sheiner et al. (2021) | To examine the role of communication sources and institutional trust as barriers and incentives as motivators of people’s attitudes toward vaccination and actual vaccination. | Israel. | Online survey (n = 484, adult participants were recruited via Blueberries). | Path analysis, structural equation modeling. |
