# Supplementary material for: Assessing the impact of COVID-19 passes and mandates on disease transmission, vaccination intention, and uptake: a scoping review
Source: BMC Public Health. 2023 Nov 17;23:2279. doi: 10.1186/s12889-023-17203-4 (PMC10656887; doi:10.1186/s12889-023-17203-4)
Supplement: Supplementary file 1 — Additional file 1. [file 12889_2023_17203_MOESM1_ESM.zip › S2 Table.docx]

**S2 Table. The impact of COVID-19 pass in each study.**

| **Study** | **Direct effect** | | **Indirect effect** | |
| --- | --- | --- | --- | --- |
|  | **+** | **-** | **+** | **-** |
| Albarracin et al. (2021) |  |  | - Participants were more willing to vaccinate in response to the required question (“Will you get the COVID-19 vaccine if it is required to work, travel, or go to school?”) than the control question (“If you could get the COVID-19 vaccine for free today, would you want to be vaccinated today?”). - Intentions were significantly stronger in the required vaccination condition than the freedom condition (M_diff_ = 0.18, SD_diff_ = 0.05, 95% CI [0.06, 0.29]) or the control for choice freedom condition (M_diff_ = 0.13, SD_diff_ = 0.05, 95% CI [0.01, 0.24]. - Intentions to vaccinate were a function of the interaction between the vaccine requirement manipulation, norms, and psychological reactance. |  |
| Alshahrani et al. (2021) |  |  | - Approximately 64% of the study participants showed an intention to accept the vaccine while 18.3% were extremely hesitant. - Factors associated with the acceptance were the source of health information about COVID-19 (OR = 1.63, 95% CI [1.07–2.47]), perception of vaccine effectiveness (OR = 7.24, 95% CI [4.58–11.45]), previous uptake of the influenza vaccine (OR = 1.62, 95% CI [1.07–2.47]), and potential vaccination mandate to travel internationally (OR = 16.52, 95% CI [10.23–26.68]). |  |
| Arif et al. (2022) |  |  |  | COVID- 19 vaccination mandates decreased the odds ratio of participant vaccination by 0.27. |
| Bennett et al. (2022) |  |  | An employer vaccine mandate leads to a net increase in the probability of vaccine uptake of 8.7 percentage points (p<0.001). |  |
| Burgio et al. (2022) | The final attack rate would decrease, increase or vary non-monotonously with respect to the mixing rate depending on the epidemic pressure, vaccine coverage and efficacy. |  |  |  |
| Cohn et al. (2022) |  |  | Cumulative adult vaccination rate for NYC increased from 72.5% to 89.4% (+16.9 percentage points [p.p.]) during the intervention period, compared with an increase from 72.5% to 83.2% (+10.7 p.p.) for the synthetic control group, which equals to a difference of 6.2 p.p. (95% CI 1.4–10.7). |  |
| Cuschieri et al. (2022) |  | - An increase in reported COVID-19 positive cases was observed at a local (host cities/regions) and national (participating and non-participating countries) from the onset of the EURO2020. - For most countries, the peak in positive cases was reached in the week of 12-18 July 2021 except for Italy, Scotland and Wales. |  |  |
| de Figueiredo et al. (2021) |  |  | - Around 78.71% (95% highest posterior density interval [76.14 to 81.04]) of the adult population who have not yet had both doses of a COVID-19 vaccine will ‘definitely’ accept a future dose, while 11.72% (10.54 to 12.83) are ‘unsure but leaning towards yes’. Around 4.51% (3.84 to 5.24) will ‘definitely not’ accept a future COVID-19 vaccine, while 5.06% (4.43 to 5.73) are ‘unsure but leaning towards no’. - Around 58.4% (56.8 to 62.6) of the adult population believe that vaccination certificates or passports is the same as requiring vaccination and 59.8% (56.8 to 62.6), believe that this is a good idea. - COVID-19 pass for international travel are less disagreeable than for domestic purposes, with net impact S_DOM_- S_INT_ = -1.99 (-3.87 to 0.13). |  |
| De Giorgio et al. (2022) |  |  | - Participants with a postgraduate degree (OR = 2.25, 95% CI [1.14–4.46]) or PhD (OR = 1.97, 95% CI [1.01–3.52], who search information on TV and radio (OR = 2.35, 95% CI [1.71–3.23]) or from general practitioner (OR = 2.53, 95% CI [1.78–3.61]) had higher odds of being vaccinated. - Around 41.6% of all participants (n=1,003) and 79.8% of vaccinated participants (n=435) found COVID-19 pass to be beneficial. |  |
| Dube et al. (2022) |  |  | - Between 9 July - 1 September 2021, the complete vaccine uptake rose from 62% to 88%. - Among unvaccinated respondents, 32% reported a positive influence of the lottery on their intention to be vaccinated against COVID-19, 39% for the vaccine passport, and 20% for both. |  |
| Ghaffarzadegan (2022) | Mandatory vaccination substantially decreased cases in institutions of higher education by 1,473 cases per 100,000 student population (95 CI: 132 - 2813). |  | - COVID-19 vaccination rates were higher in institutions that mandated it. |  |
| Hohenegger et al. (2022) | An efficiency of a vaccine-only COVID-19 pass of roughly 20-40% is strong enough to completely suppress a potential new wave. | The efficacy of a vaccine-testing COVID-19 pass needs to roughly be twice as high to produce the same reduction of infections as a vaccine-only COVID-19 pass. |  |  |
| Howard-Williams et al. (2022) |  |  | - Around 634,831 first doses (11.5%) were associated with the mandate announcement. - First-dose administration coverage among 13 jurisdictions increased starting at 3 weeks after the mandate announcement, with statistically significant differences of 0.20, 0.33, 0.39, 0.45, 0.49, and 0.59 percentage points higher than the referent category coverage of 62.9%. |  |
| Hubble et al. (2022) |  |  | - About 74.7% of the total respondents reported receiving the COVID-19 vaccination - Predictors of vaccination included belief in vaccine safety (odds ratio [OR] 5.5, p-value <0.001) and effectiveness (OR 4.6, p-value <0.001); importance of vaccination to protect patients (OR 15.5, p-value <0.001); perceived personal risk of infection (OR 1.8, p-value = 0.04); previous receipt of influenza vaccine (OR 2.5, p-value = 0.003); and sufficient knowledge to make an informed decision about vaccination (OR 2.4, p-value = 0.024) | Only 18.7% of the total respondents supported mandatory vaccination for EMS professionals |
| Iwu et al. (2022) |  |  |  | - The prevalence of vaccine hesitancy was 35.4%. - More than half of the respondents (67.4%) would not encourage vaccination mandates. |
| Juarez et al. (2022) | The number of COVID-19 cases in counties that implemented vaccine or test passport mandates was decreased by 19.0% |  | - The vaccination rate increased by 1.41% in counties that implemented mandates. - Businesses that fell under the mandates had a significantly (6.7%, 95%Cl: 5.7–6.9%) lower number of customers, but later on, the number converged towards the level of that of non-impacted businesses. |  |
| Karaivanov et al. (2022) |  |  | - As of 31 October 2021, the vaccination mandate increased vaccine uptake up to 5 percentage points (p.p.) (90% CI [3.9–5.8]) for Canadian provinces, adding up to 979,000 (425,000–1,266,000) first doses in total for Canada (5 to 13 weeks after the provincial mandate announcements). - In France, the uptake is increased up to 8 p.p. (90% CI [4.3–11]) within 16 weeks post-announcement, 12 p.p. (90% CI [5–15]) in Italy within 14 weeks post-announcement, 4.7 p.p. (90% CI [4.1–5.1]) in Germany within 11 weeks post-announcement. |  |
| Kaufman et al. (2022) |  |  | - More than half of all HCWs (57%, 1754/3058) said they would be more likely to get vaccinated if required by their employer. - Of those who did not intend to get the vaccine, 23% (159/676) indicated that they would be more likely to get vaccinated if their employer required it. |  |
| Kelekar et al. (2021) |  |  | - Medical students (MS) were 2.7 times more likely than dental students (DS) to receive the vaccine (odds ratio, 2.74; 95% CI, 1.76 to 4.31; p-value < .0001). - MS were more likely to agree with mandates and trust information about the vaccines. - After controlling for demographic variables, experience with COVID-19, and personal vaccination behaviors, those who thought the COVID-19 vaccine was important to them as health care professionals, trusted COVID-19 information received from public health experts, and thought the COVID-19 vaccination should be mandatory for the general public were statistically significantly more likely to report willingness to get the COVID-19 vaccine (p-value < .01). |  |
| Klüver et al. (2021) |  |  | - Among all participants, the lowest treatment effect was found in low financial incentive (1 p.p.) followed by higher financial incentives (2.2 p.p.), additional freedoms (2.5 p.p.), vaccinations at the local doctor (3 p.p.). - Combination of incentives increased vaccine acceptance up to 13 p.p. from a baseline of 40% among undecided participants. |  |
| Kuznetsova et al. (202) |  |  | - The introduction of COVID-19 certificates was associated with a significant immediate or gradual increase in daily administered vaccine doses in all the countries included in the study, up to 117,617 doses gained per million per month. - The effect of mandatory vaccination for all or some groups of the population varied from a continuous decrease in daily administered doses (332 per million capita per day), no significant effect, or a delayed or temporary increase (1489 per million capita per day). |  |
| Ledda et al. (2021) |  |  | - Higher rates of acceptance of mandatory vaccinations for healthcare professionals were expressed for those caring for immunocompromised patients. - There is a statistically significant difference for gender, profession, and education in the intention to get a COVID-19 vaccine. |  |
| Maltezou et al (2021) |  |  | - Around 776 participants (49.8%) favored mandatory vaccinations policies. - Male gender, being a physician, being completely vaccinated against hepatitis B, have been vaccinated against pandemic A (H1N1) in 2009-2010, belief that COVID-19 vaccination should be mandatory for HCP, and increased confidence in vaccines in general during the ongoing COVID-19 pandemic were positively associated with intention to get vaccinated. |  |
| McGarry et al. (2022) |  |  | - Mean staff vaccine coverage was estimated to increase by 5.4 percentage points (95% CI 1.1-9.8) in mandate states without a test-out option and 2.2 percentage points (95% CI 0.8-3.5) in mandate states with a test-out option following mandate announcement. |  |
| Mills and Rüttenauer (2022) |  |  | - COVID-19 certification led to increased vaccinations 20 days before implementation in anticipation, with a lasting effect up to 40 days after. - Countries with below average pre-intervention uptake had a more pronounced increase in daily vaccinations compared to those with average or high uptake (for example in France, the vaccine uptake was increased from 3,761,440 (95% CI [3,355,761-–4,979,952]) doses before mandatory certification to 4,874,857 (95% CI [2,563,396–-7,711,769]) doses after certification. - Increase in uptake was highest for people younger than 30 years after the introduction of certification (up to 10,000 daily doses per million population). |  |
| Moccia et al. (2022) |  |  | Around 85.4% of the respondents stated that “Getting vaccinated is the only way to return to our life, to normal” and 4.9% stated “Vaccinating is the only way to get the Green Pass”. |  |
| Mouter et al. (2022) |  |  | All respondents had a positive preference for the vaccination passport policies and a negative preference for the “mandatory testing at own cost if does not get vaccinated” or the “pay EUR 250 if does not get vaccinated” policies. |  |
| Mustapha et al. (2021) |  |  | - Around 52.3% of the respondents were willing to take the vaccine if mandated by their institution heads. - Older age, mandate by heads of the institution, trust in the government and readiness to pay for the vaccine were associated with acceptance of the vaccine |  |
| Okamoto et al. (2022) |  |  | - Around 45% of all the vaccine- hesitant respondents intended to accept vaccination when all public health restrictions were relaxed, while 18% intended to do so if no restrictions were relaxed. - Vaccine passports, which allow citizens freedom in their daily lives, could increase vaccine acceptance when they are imposed with public health restrictions due to the COVID-19 pandemic, particularly for younger people. |  |
| Oliu-Barton et al. (2022) | - The expected number of hospital admissions without COVID-19 pass would have been 31.3% higher in France, 5.0% higher in Germany, and 15.5% higher in Italy. - The expected number of deaths would have been 31.7% higher in France, 5.6% higher in Germany, and 14.0% higher in Italy. |  | - The announcement of COVID certificates during summer 2021 were associated with increased vaccine uptake in France (13.0 p.p., 95% CI [9.7–14.9)), Germany (6.2 p.p. 95% CI [2.6–6.9]), and Italy (9.7 p.p., 95% CI [5.4–12.3]). - Gross domestic product (GDP) losses of EUR 6.0 billion in France, EUR1.4 billion in Germany, and EUR 2.1 billion in Italy could be prevented. |  |
| Peruch et al. (2022) |  |  | - About 96.1% (n = 125) of the respondents had been vaccinated. - Around 5.4% stated that they agreed to be vaccinated exclusively because of the sanctions provided for by the legislation. | - Despite the high rate of response to vaccination, 17.7% of the respondents did not agree with its mandatory nature. |
| Porat et al. (2021) |  |  |  | Negative correlation between autonomy frustration and vaccine acceptance (r = −0.37, 95% CI [−0.32, −0.41]). The more participants were against vaccine passports, the more autonomy frustration they reported. |
| Radic et al. (2022) |  |  | - Mass media coverage has a positive impact on the attitude toward a COVID-19 vaccine certificate (β=0.523, p< 0.001). - Attitude towards a COVID-19 vaccine certificate (β =0.434, p< 0.001), subjective norm towards the COVID-19 vaccine (β =0.701, p< 0.001), and previous travel experience (β =0.137, p< 0.001) have a positive impact on the behavioral intention to take the COVID-19 vaccine before international travel. | Travel motivation has a negative impact on the attitude toward a COVID-19 vaccine certificate (β =-0.199, p< 0.001). |
| Raja et al. (2022) |  |  | - Vaccine acceptance among the respondents was 55.8% - Around 58.7% of all respondents were willing to accept the vaccine for travel reasons. |  |
| Ramos et al. (2022) | The incidence rate was lower in the intervention group (attendees) at 7 days (10.6 per 100,000 person-days, 95% CI [0.3–58.9] versus 21.2 per 100,000 person-days, 95% CI [7.8–46.1]) and 14 days (15.9 per 100,000 person-days, 95% CI [3.3–46.4] versus 17.7 per 100,000 person-days, 95% CI [8.5–32.5]) of follow-up. This difference was not statistically significant. |  |  |  |
| Reno et al. (2022) | After the implementation of Green Pass (23 July 2021), the number of new cases, hospitalizations, and deaths were considerably lower in this fourth wave compared to the previous waves. |  | The vaccination rate increased immediately from 50% to 60%. |  |
| Rosen et al. (2021) |  |  | By March 31, 73% of the population age 20–29 year had been vaccinated, despite an initially slow uptake and despite the initial hesitancy prevalent among this age group. It is very likely that the Green Pass program have been particularly influential in this age group. |  |
| Saban et al. (2021) |  |  | - After the exemption from quarantine for vaccinated persons on 17 January 2021, there was a peak in second dose vaccinations up to over 200,000 doses per day. - Another peak was observed on 21 February when the Green Pass was officially implemented. |  |
| Sargent et al. (2022) |  |  | Among working unvaccinated respondents (n = 2,135), 32.1% said that they would get vaccinated in response to a work requirement, 42.2% said they would not get vaccinated, and 25.7% reported that they were unsure if they would get vaccinated. |  |
| Shmueli (2022) |  |  |  | Neither financial incentives, such as monetary rewards (OR = 1.07, 95% CI [0.76, 1.50]) or monetary penalties (OR = 0.98, 95% CI [0.71,1.36]), nor non-financial incentives, such as the “green pass” (OR = 1.12, 95% CI [0.82,1.55]) were found to be significant predictors of the intention to get vaccinated. |
| Syme et al. (2022) |  |  | Staff COVID-19 vaccination rates in Mississippi increased from 43.0% before the vaccinate-or-test-out mandate to 51.3%. |  |
| Tchepmo Djomegni et al. (2021) | - When 1 out of 1000 susceptible individuals are (cumulatively) "protected", then the pandemic will reach its peak approximately after 150 days, which is earlier than without "protected class". - There will be about 400,000 active case in this period. After some days, most of the susceptible will be "protected” and the population could be disease free over time. |  |  |  |
| Walkowiak et al. (2021) |  |  | - The mandate resulted in a 12.34% increase in the vaccination rate in Lithuania. - The vaccination rate increased considerably in all age group: 43.1% in age group 18–24 years, 45.1% in age 25–49 years, 41.3% in age 50–59 years, 40.2% in age 60–69 years, and 20.3% in 70+ year. |  |
| Wong et al. (2021) |  |  | The highest score of facilitation measures was for “vaccine passports for overseas travel” (4.44, 95% C.I. [4.18, 4.71]). |  |
| Zimand-Sheiner et al. (2021) |  |  | - Incentives positively affects attitude toward vaccination (β = 0.417, p<0.001). - The use of incentives can reduce the institutional mistrust effect and enhance vaccination attitude and actual vaccination (β = −0.127, p = 0.014) |  |
