# Supplementary material for: Assessing the impact of COVID-19 passes and mandates on disease transmission, vaccination intention, and uptake: a scoping review
Source: BMC Public Health. 2023 Nov 17;23:2279. doi: 10.1186/s12889-023-17203-4 (PMC10656887; doi:10.1186/s12889-023-17203-4)
Supplement: Supplementary file 1 — Additional file 1. [file 12889_2023_17203_MOESM1_ESM.zip › S3 Table.docx]

**S3 Table. Quality assessment of studies included in the synthesis.**

**Cross-sectional studies**

| **Study** | **Item** | | | | | | | | **%** | **Quality** |
| --- | --- | --- | --- | --- | --- | --- | --- | --- | --- | --- |
|  | **1** | **2** | **3** | **4** | **5** | **6** | **7** | **8** |  |  |
| Albarracin et al. (2021) | Y | Y | Y | Y | N | N | Y | Y | 75 | Moderate |
| Alshahrani et al. (2021) | Y | Y | Y | Y | Y | Y | Y | Y | 100 | Good |
| Arif et al. (2022) | Y | Y | Y | Y | Y | N | Y | Y | 87.5 | Good |
| Bennett et al. (2022) | N | Y | Y | Y | Y | Y | Y | Y | 87.5 | Good |
| Cuschieri et al. (2022) | Y | Y | Y | Y | N | N | Y | Y | 75 | Moderate |
| de Figueiredo et al. (2021) | N | Y | Y | Y | Y | Y | Y | Y | 87.5 | Good |
| De Giorgio et al. (2022) | Y | Y | Y | Y | Y | Y | Y | Y | 100 | Good |
| Dube et al. (2022) | N | Y | Y | Y | Y | N | Y | Y | 75 | Moderate |
| Ghaffarzadegan (2022) | Y | N | N | Y | N | N | Y | Y | 50 | Moderate |
| Howard-Williams et al. (2022) | Y | Y | Y | Y | NA | NA | Y | Y | 100 | Good |
| Hubble et al. (2022) | N | Y | U | U | Y | Y | U | Y | 50 | Fair |
| Iwu et al. (2022) | Y | Y | Y | Y | N | N | Y | Y | 75 | Moderate |
| Juarez et al. (2022) | Y | Y | Y | Y | Y | Y | Y | Y | 100 | Good |
| Kaufman et al. (2022) | Y | Y | Y | Y | Y | Y | Y | Y | 100 | Good |
| Kelekar et al. (2021) | Y | Y | Y | Y | Y | Y | Y | Y | 100 | Good |
| Klüver et al. (2021) | Y | Y | Y | Y | N | N | Y | Y | 75 | Moderate |
| Ledda et al. (2021) | Y | Y | Y | Y | N | N | Y | Y | 75 | Moderate |
| Maltezou et al (2021) | Y | Y | Y | Y | N | N | Y | Y | 75 | Moderate |
| Moccia et al. (2022) | Y | Y | Y | Y | N | N | Y | U | 63 | Moderate |
| Mouter et al. (2022) | Y | Y | Y | Y | Y | Y | Y | Y | 100 | Good |
| Mustapha et al. (2021) | Y | Y | Y | Y | Y | Y | Y | Y | 100 | Good |
| Okamoto et al. (2022) | Y | Y | Y | NA | Y | Y | Y | Y | 100 | Good |
| Peruch et al. (2022) | Y | Y | Y | NA | N | N | Y | U | 57.14 | Moderate |
| Porat et al. (2021) | U | Y | Y | NA | Y | Y | Y | Y | 85.71 | Good |
| Radic et al. (2022) | Y | Y | Y | Y | N | U | Y | Y | 75 | Moderate |
| Raja et al. (2022) | Y | Y | Y | NA | N | U | Y | Y | 71.43 | Moderate |
| Reno et al. (2022) | Y | Y | NA | NA | NA | NA | Y | Y | 100 | Good |
| Rosen et al. (2021) | Y | Y | NA | NA | NA | NA | Y | Y | 100 | Good |
| Saban et al. (2021) | Y | Y | NA | NA | NA | NA | Y | Y | 100 | Good |
| Sargent et al. (2022) | Y | Y | N | NA | U | Y | Y | Y | 71.43 | Moderate |
| Shmueli (2022) | Y | Y | N | NA | U | Y | Y | Y | 71.43 | Moderate |
| Syme et al. (2022) | Y | Y | NA | NA | NA | NA | Y | Y | 100 | Good |
| Walkowiak et al. (2021) | Y | Y | NA | NA | NA | NA | Y | Y | 100 | Good |
| Wong et al. (2021) | Y | Y | Y | NA | U | Y | Y | U | 71.43 | Moderate |
| Zimand-Sheiner et al. (2021) | Y | Y | Y | NA | U | Y | Y | Y | 71.43 | Moderate |

Y = yes; N = no; U = unclear, NA = not applicable.

The item corresponds to the following questions from the JBI critical appraisal tool for cross-sectional studies: 1 = Were the criteria for inclusion in the sample clearly defined?; 2 = Were the study subjects and the setting described in detail?; 3 = Was the exposure measured in a valid and reliable way?; 4 = Were objective, standard criteria used for measurement of the condition?; 5 = Were confounding factors identified?; 6 = Were strategies to deal with confounding factors stated?; 7 = Were the outcomes measured in a valid and reliable way?; 8 = Was appropriate statistical analysis used?

Quality: Fair when <50% of the items given a rating of yes; moderate when 51-80% of the items given a rating of yes; good when >80% of items given a rating of yes. Items with a rating of NA were excluded from the score calculation.

**Randomized controlled trial**

| **Study** |  |  |  | **Item** | | | | | | | | | | **%** | **Quality** |
| --- | --- | --- | --- | --- | --- | --- | --- | --- | --- | --- | --- | --- | --- | --- | --- |
|  | **1** | **2** | **3** | **4** | **5** | **6** | **7** | **8** | **9** | **10** | **11** | **12** | **13** |  |  |
| Klüver et al. (2021) | Y | U | U | NA | NA | NA | NA | Y | Y | Y | Y | Y | Y | 78 | Moderate |

Y = yes; N = no; U = unclear, NA = not applicable.

The item corresponds to the following questions from the JBI critical appraisal tool for cohort studies: 1 = Was true randomization used for assignment of participants to treatment groups?; 2 = Was allocation to treatment groups concealed?; 3 = Were treatment groups similar at the baseline?; 4 = Were participants blind to treatment assignment?; 5 = Were those delivering treatment blind to treatment assignment?; 6 = Were outcomes assessors blind to treatment assignment?; 7 = Were treatment groups treated identically other than the intervention of interest?; 8 = Was follow up complete and if not, were differences between groups in terms of their follow up adequately described and analyzed?; 9 = Were participants analyzed in the groups to which they were randomized?; 10 = Were outcomes measured in the same way for treatment groups?; 11 = Were outcomes measured in a reliable way?; 12 = Was appropriate statistical analysis used?; 13 = Was the trial design appropriate, and any deviations from the standard RCT design (individual randomization, parallel groups) accounted for in the conduct and analysis of the trial?

Quality: Fair when <50% of the items given a rating of yes; moderate when 51-80% of the items given a rating of yes; good when >80% of items given a rating of yes. Items with a rating of NA were excluded from the score calculation.

**Cohort studies**

| **Study** | **Item** | | | | | | | | | | | **%** | **Quality** |
| --- | --- | --- | --- | --- | --- | --- | --- | --- | --- | --- | --- | --- | --- |
|  | **1** | **2** | **3** | **4** | **5** | **6** | **7** | **8** | **9** | **10** | **11** |  |  |
| McGarry et al. (2022) | Y | Y | Y | Y | Y | N | Y | Y | Y | NA | Y | 90 | Good |

Y = yes; N = no; U = unclear, NA = not applicable.

The item corresponds to the following questions from the JBI critical appraisal tool for cohort studies: 1 = Were the two groups similar and recruited from the same population?; 2 = Were the exposures measured similarly to assign people to both exposed and unexposed groups?; 3 = Was the exposure measured in a valid and reliable way?; 4 = Were confounding factors identified?; 5 = Were strategies to deal with confounding factors stated?; 6 = Were the groups/participants free of the outcome at the start of the study (or at the moment of exposure)?; 7 = Were the outcomes measured in a valid and reliable way?; 8 = Was the follow up time reported and sufficient to be long enough for outcomes to occur?; 9 = Was follow up complete, and if not, were the reasons to loss to follow up described and explored?; 10 = Were strategies to address incomplete follow up utilized?; 11 = Was appropriate statistical analysis used?

Quality: Fair when <50% of the items given a rating of yes; moderate when 51-80% of the items given a rating of yes; good when >80% of items given a rating of yes. Items with a rating of NA were excluded from the score calculation.

**Quasi-experimental studies**

| **Study** | **Item** | | | | | | | | | **%** | **Quality** |
| --- | --- | --- | --- | --- | --- | --- | --- | --- | --- | --- | --- |
|  | **1** | **2** | **3** | **4** | **5** | **6** | **7** | **8** | **9** |  |  |
| Ramos et al. (2022) | Y | Y | U | Y | Y | U | Y | Y | Y | 77.77 | Moderate |

Y = yes; N = no; U = unclear, NA = not applicable.

The item corresponds to the following questions from the JBI critical appraisal tool for quasi-experimental studies: 1 = Is it clear in the study what is the ‘cause’ and what is the ‘effect’ (i.e. there is no confusion about which variable comes first)?; 2 = Were the participants included in any comparisons similar? 3 = Were the participants included in any comparisons receiving similar treatment/care, other than the exposure or intervention of interest?; 4 = Was there a control group?; 5 = Were there multiple measurements of the outcome both pre and post the intervention/exposure?; 6 = Was follow up complete and if not, were differences between groups in terms of their follow up adequately described and analyzed?; 7 = Were the outcomes of participants included in any comparisons measured in the same way?; 8 = Were outcomes measured in a reliable way?; 9 = Was appropriate statistical analysis used?

Quality: Fair when <50% of the items given a rating of yes; moderate when 51-80% of the items given a rating of yes; good when >80% of items given a rating of yes. Items with a rating of NA were excluded from the score calculation.

**Mathematical modeling studies**

| **Study** | **Item** | | | | | | | | | | | | | | | | | | | | **%** | | **Quality** | |
| --- | --- | --- | --- | --- | --- | --- | --- | --- | --- | --- | --- | --- | --- | --- | --- | --- | --- | --- | --- | --- | --- | --- | --- | --- |
|  | **1** | **2** | **3** | **4** | **5** | **6** | **7** | **8** | **9** | **10** | **11** | **12** | **13** | **14** | **15** | **16** | **17** | **18** | **19** |  | |  | |  |
| Cohn et al. (2022) | Y | Y | Y | Y | Y | Y | Y | Y | N | Y | Y | Y | NA | N | Y | N | Y | Y | N | 77 | | Moderate | |  |
| Burgio et al. (2022) | Y | Y | Y | NA | N | Y | N | Y | Y | Y | NA | Y | NA | N | Y | N | Y | Y | N | 69 | | Moderate | |  |
| Hohenegger et al. (2022) | Y | Y | Y | NA | Y | Y | Y | Y | N | Y | NA | Y | NA | N | Y | N | N | N | N | 63 | | Moderate | |  |
| Karaivanov et al. (2022) | Y | Y | Y | Y | Y | N | Y | Y | N | Y | Y | N | Y | Y | N | N | Y | Y | N | 68 | | Moderate | |  |
| Kuznetsova et al. (2022) | N | N | Y | Y | Y | N | Y | Y | N | N | N | N | Y | N | Y | N | N | Y | N | 47 | | Fair | |  |
| Mills and Rüttenauer (2022) | Y | Y | Y | Y | Y | Y | Y | Y | N | Y | Y | N | Y | Y | Y | N | N | Y | Y | 79 | | Moderate | |  |
| Oliu-Barton et al. (2022) | N | Y | Y | Y | Y | Y | Y | Y | Y | Y | Y | Y | N | Y | Y | N | Y | Y | Y | 84.21 | | Good | |  |
| Tchepmo Djomegni et al. (2021) | Y | Y | Y | N | Y | Y | Y | Y | N | Y | Y | N | U | N | Y | N | N | Y | N | 57.9 | | Moderate | |  |

Y = yes; N = no; U = unclear, NA = not applicable.

The item corresponds to the following questions from the EPIFORGE 2020 checklist: 1 = Describe the study as forecast or prediction research in at least the title or abstract; 2 = Define the purpose of study and forecasting targets; 3 = Fully document the methods; 4 = Identify whether the forecast was performed prospectively, in real time, and/or retrospectively; 5 = Explicitly describe the origin of input source data, with references; 6 = Provide source data with publication, or document reasons as to why this was not possible; 7 = Describe input data processing procedures in detail; 8 = State and describe the model type, and document model assumptions, including references; 9 = Make the model code available, or document the reasons why this is not possible; 10 = Describe the model validation, and justify the approach; 11 = Describe the forecast accuracy evaluation method used, with justification; 12 = Where possible, compare results to a benchmark or other comparator model, with justification of comparator choice; 13 = Describe the forecast horizon, with justification of its length; 14 =  Present and explain uncertainty of forecasting results; 15 = Briefly summarize the results in nontechnical terms, including a nontechnical interpretation of forecast uncertainty; 16 = If results are published as a data object, encourage a time-stamped version number; 17 = Describe the weaknesses of the forecast, including weaknesses specific to data quality and methods; 18 = If the forecast research is applicable to a specific epidemic, comment on its potential implications and impact for public health action and decision-making; 19 = If the forecast research is applicable to a specific epidemic, comment on how generalizable it may be across populations.

Quality: Fair when <50% of the items given a rating of yes; moderate when 51-80% of the items given a rating of yes; good when >80% of items given a rating of yes. Items with a rating of NA were excluded from the score calculation.
